# The eco-friendly spectrophotometric methods for duloxetine and amitriptyline quantification using eosin Y: content uniformity and greenness evaluation

**DOI:** 10.1186/s13065-025-01428-y

**Published:** 2025-03-08

**Authors:** Al Amir S. Zaafan, Hadeer A. Elhamdy

**Affiliations:** Pharmaceutical Analytical Chemistry, Faculty of Pharmacy, Sohag University, Sohag, 82524 Egypt

**Keywords:** Duloxetine, Amitriptyline, Eosin Y, Content uniformity, Greenness evaluation

## Abstract

Straightforward, eco-friendly, quick, and sensitive spectrophotometric procedures were created and proven to be effective for determining the amount of duloxetine and amitriptyline in bulk and pharmaceutical dosage forms. The basis of the suggested procedures was the formation of an ion association complex in an aqueous buffered solution containing duloxetine and amitriptyline with eosin Y. The resulting compound displayed absorption peaks at 546 nm under optimum circumstances. With a linear relationship and a good correlation value of 0.9996 for DLX and 0.9997 for AMT, the calibration plots were rectilinear over the concentration range of 0.5–8 µg mL^− 1^ for DLX and 1–7 µg mL^− 1^ for AMT. The quantitation limits were 0.48 and 0.49 µg mL^− 1^ for DLX and AMT, respectively, whereas the detection limits were 0.16 µg mL^− 1^ for both drugs. The research process has been optimized with respect to the many experimental parameters. The approaches were assessed in accordance with ICH guidelines. The suggested approaches were successfully used to analyze pharmaceutical formulations, including the cited medications. Additionally, the recommended methods performed admirably when used to assess content uniformity. The proposed method is highly green as water was used as the solvent. Utilizing four metric tools called the NEMI, Eco-Scale, GAPI, and AGREE, the established techniques’ environmental impact was assessed. Also, the practicality (blueness) of procedures was assessed using a recently developed metric called the Blue Applicability Grade Index (BAGI).

## Introduction

**Fig. 1:**
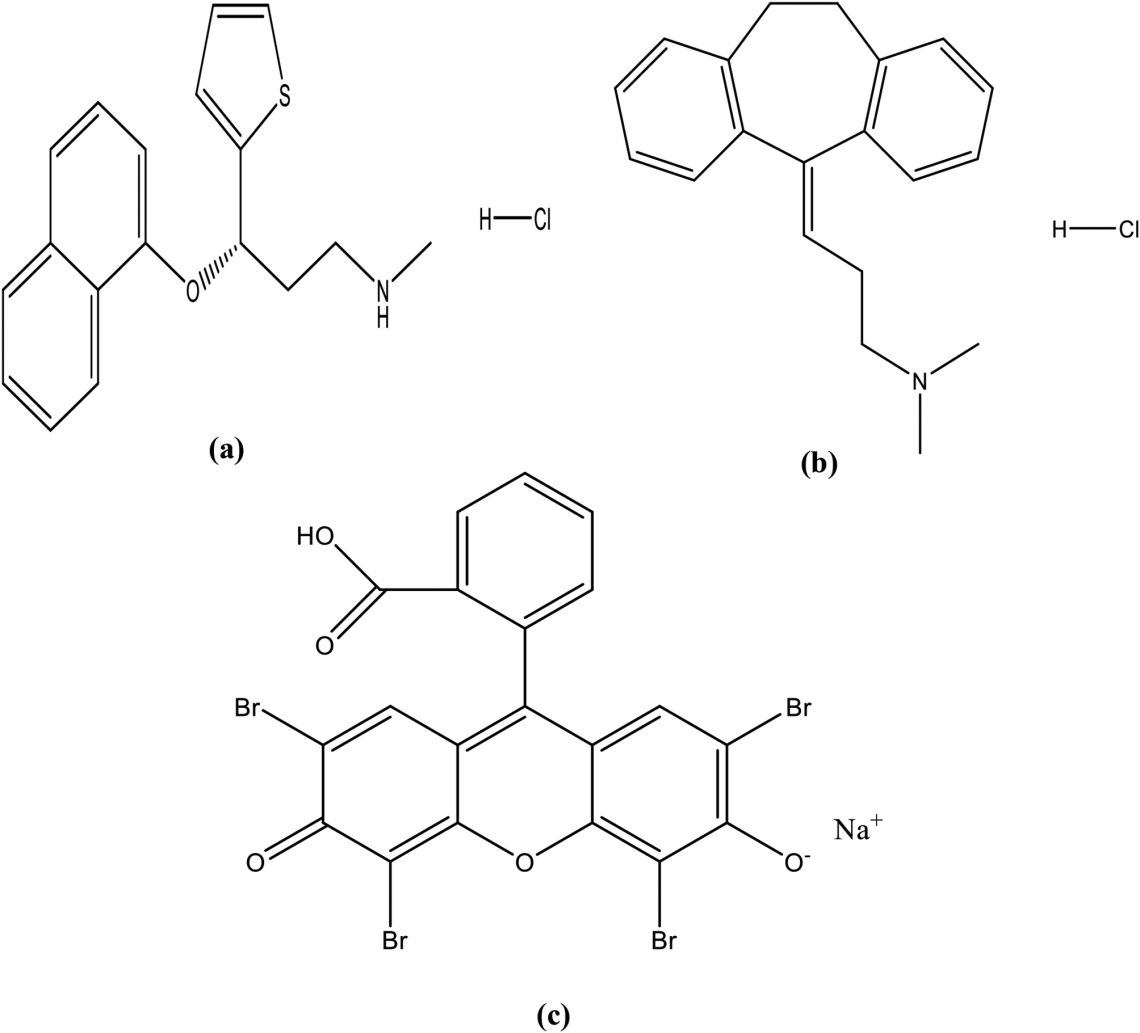
Chemical structures of Duloxetine hydrochloride (**a**), Amitriptyline hydrochloride (**b**) and Eosin Y (**c**)

Duloxetine hydrochloride (DLX, Fig. 1) is N-methyl-3-napthalen-1-oxy-3-thiophen-2-yl-1-amine. Serious depressive conditions are treated with DLX, a selective serotonin and norepinephrine reuptake inhibitor [1]. It works effectively as both a norepinephrine and serotonin reuptake blocker. Dopaminergic, cholinergic, histaminergic, glutamate, adrenergic, opioid, and GABA receptors are not significantly stimulated by DLX [2]. Major depressive illness, anxiety disorders, fibromyalgia, peripheral neuropathy pain in individuals with diabetes or pain caused by treatment with chemotherapy are among the conditions for which it is recommended [3, 4], and incontinence of urination due to stress, as well [5]. Compared to other antidepressants, DLX has numerous benefits, including increased safety, tolerance, efficacy, and fewer side effects. It also has dual inhibitory qualities and a lesser affinity for neural receptors [6]. In order to determine DLX, various methods have been reported, including spectrophotometry [7, 8, 9, 10], spectrofluorimetry [11, 12], TLC [13, 14, 15], HPLC [16, 17, 18, 19], GC [20] and electrochemical methods [21, 22]. Amitriptyline hydrochloride, (AMT, Fig. 1) is 3-(10,11-dihydro-5 H-dibenzo cycloheptene-5-ylidene)-N, N-dimethyl propan-1-amine hydrochloride. Manic depression, anxiety, and involutional melancholia can all be effectively treated with AMT [23]. A multitude of psychiatric diseases are treated with the medication AMT. It is one of the most widely used tricyclic antidepressants, it inhibits the membrane pump pathway that allows serotonin and norepinephrine to enter adrenergic and serotonergic neurons [24, 25]. A variety of analytical methods have been reported such as spectrophotometry [26, 27, 28], spectrofluorimetry [1, 29, 30, 31, 32], TLC [33, 34, 35, 36], HPLC [37, 38, 39, 40], UPLC [41] GC/MS [42], capillary electrophoresis [43] and electrochemical methods [44, 45, 46, 47, 48, 49, 50]. The previously described techniques do, however, have certain drawbacks in terms of selectivity, sensitivity, cost, duration, and environmental friendliness. The majority of previous techniques for determining AMT or DLX are dependent on HPLC, which makes extensive use of extremely pure organic solvents. These techniques are more expensive and have poor environmental safety, particularly if a mass detector is used. No simple visible-spectrophotometric methods for the determination of DLX or AMT based on ion pair complex are developed, while the reported spectrophotometric methods need heating [27] or extraction with organic solvent [26]. Consequently, a cheap and more sensitive spectrophotometric approach could be developed for the determination of DLX and AMT. This inspired the authors to design a new spectrophotometric method that is direct, rapid, simple and accurate for DLX and AMT determination. The novelty of the current work comes from the fact that, to date, there is no published simple visible-spectrophotometric method for determination of DLX or AMT based on ion pair complex with a dye. The proposed approach focuses on the development of an association complex between the medications under investigation and eosin Y. Eosin Y (Fig. 1) is an ion-pairing reagent that can also be used as a fluorogenic substance. Analysis of drugs can be effectively conducted by the use of the donor-acceptor reaction, which involves the transfer of charges and the production of ion pair complexes [51, 52, 53]. In acidic media, the amino group in the medications under investigation is protonated, producing a cationic molecule suitable for interaction with the negatively charged dye constituent. The resulting ion pair was readily dissolved in water, removing the requirement for extraction using potentially dangerous organic solvents and enabling direct observations. The proposed technique offered a straightforward, precise, and extraction-free spectrophotometric technique for quantifying the investigated drugs in pharmaceutical formulations. Spectroscopic methods are characterized by simplicity [54]. Other benefits of the suggested process include an inexpensive reagent and a device that is simple, affordable, and accessible in the majority of quality control departments. The method’s analytical performance was evaluated in accordance with the ICH strategies.

Since this is the first visible-spectrophotometric approach based on an ion pair complex formation with a dye that doesn’t require extraction, the present study is innovative. The innovation also involves the use of a simple extraction process, since it utilizes a single, straightforward procedure for sample preparation rather than using a number of complicated steps. The suggested method has been effectively applied to verify the content uniformity of medication tablet formulations because of its simple manipulation process. Additionally, the procedure is extremely environmentally friendly and compliant with green chemistry standards because water serves as the diluting solvent for the reaction. The method’s greenness was evaluated using a number of tools, namely, NEMI, Eco-scale, Green Analytical Procedure Index (GAPI), and the Analytical Greenness metric approach and software (AGREE). Also, the sustainability of the proposed method was also assessed using the Blue Applicability Grade Index (BAGI), a recently advanced metric for assessing the blueness of approaches.

## Experimental

### Apparatus

A T80 double beam UV–VIS spectrophotometer (PG instruments, Leicestershire, UK) connected to UV Win software was used for the spectrophotometric measurements. The measurements were taken in one-centimeter quartz cells. A Jenway 3510 pH meter (Staffordshire, UK) was used for controlling the pH. Aquatron water still a4000d, double-distilled (Cole-Parmer, Staffordshire, UK).

### Materials and reagents

The entire methodology was completed with doubly distilled water and reagents of the analytical grade. The pharmaceutical company Mash Premiere (Badr City, Cairo, Egypt) was kindly provided DLX. AMT was attained from (El-kahira pharmacological and chemical Co., Cairo, Egypt). Cymbatex 20 mg capsules and tryptizol 10 mg tablets were bought from the local market. A 1 × 10^− 3^ M aqueous solution of eosin Y (Merck, Darmstadt, Germany) was made with distilled water. El Nasr Pharmaceutical Chemical Co. was the manufacturer of the citric and phosphoric acids, NaOH, acetic acid and sodium acetate (Cairo, Egypt). Teorell-Stenhagen buffer was created by mixing 0.1 M citric acid, 0.1 M NaOH, and 0.1 M phosphoric acid in different ratios to provide 0.1 M solutions with a range of pH values while acetate buffer solution was organized by combining the appropriate volumes of 0.1 M acetic acid and 0.1 M sodium acetate. A pH meter was used to adjust the necessary pH, the pH range was 3.2–4.6.

### Standard solutions

To produce the stock solutions, 10.0 mg of each drug was dissolved in 100 mL of distilled water to obtain 100.0 µg/mL. After that, working solutions employing concentrations around 0.5–8 µg mL^− 1^ for DLX and 1–7 µg mL^− 1^ for AMT were prepared by diluting the resulting solution using the same solvent. If this solution was refrigerated and protected from light, it persisted stable for at least a week.

### General assay procedure

One milliliter of standard solution was transferred into a 10-mL volumetric flask to get a final concentration range of 0.5–8 µg mL^− 1^ for DLX and 1–7 µg mL^− 1^ for AMT, Regarding DLX, the following reagents were added: 1 mL of 0.1 M Teorell-Stenhagen buffer (pH 3.7) and 1.4 mL eosin Y (1 × 10^− 3^ M). On the other hand, 0.6 mL of 0.1 M acetate buffer (pH 3.8) and 1.6 mL eosin Y (1 × 10^− 3^ M) were added in the case of AMT. The volume was then completed to the mark with distilled water. The absorption amplitude was subsequently determined at 546 nm. A blank assay was set up using the identical process, but no drug solution was added. Plotting the estimated absorbance versus the drug’s actual concentration.

### Preparation of pharmaceutical dosage forms

Ten capsules containing DLX or ten tablets containing AMT were carefully inserted into a mortar, and then the powder was formed via fine grinding. A quantity of the powder equivalent to 20 mg DLX or 10 mg AMT was weighed accurately, transferred to a volumetric glass flask (100 mL), and extracted with methanol via sonication for 30 min. Using the same solvent, the volume was completed to the mark. After filtration of the solution, the initial part of the filtrate was rejected. An aliquot of the filtrate was substantially diluted with the same solvent to yield a solution whose final concentration fell throughout the necessary range. The quantity of the drugs in the final solution was examined using the standard procedure.

### Content uniformity testing

The content uniformity of DLX capsules or AMT tablets was tested in accordance with USP requirements [55]. Following the steps described under “Preparation of pharmaceutical dosage forms”, a separate examination of each capsule or tablet was conducted in order to assess the homogeneity of the contents of ten capsules or tablets.

## Results and discussion

**Fig. 2:**
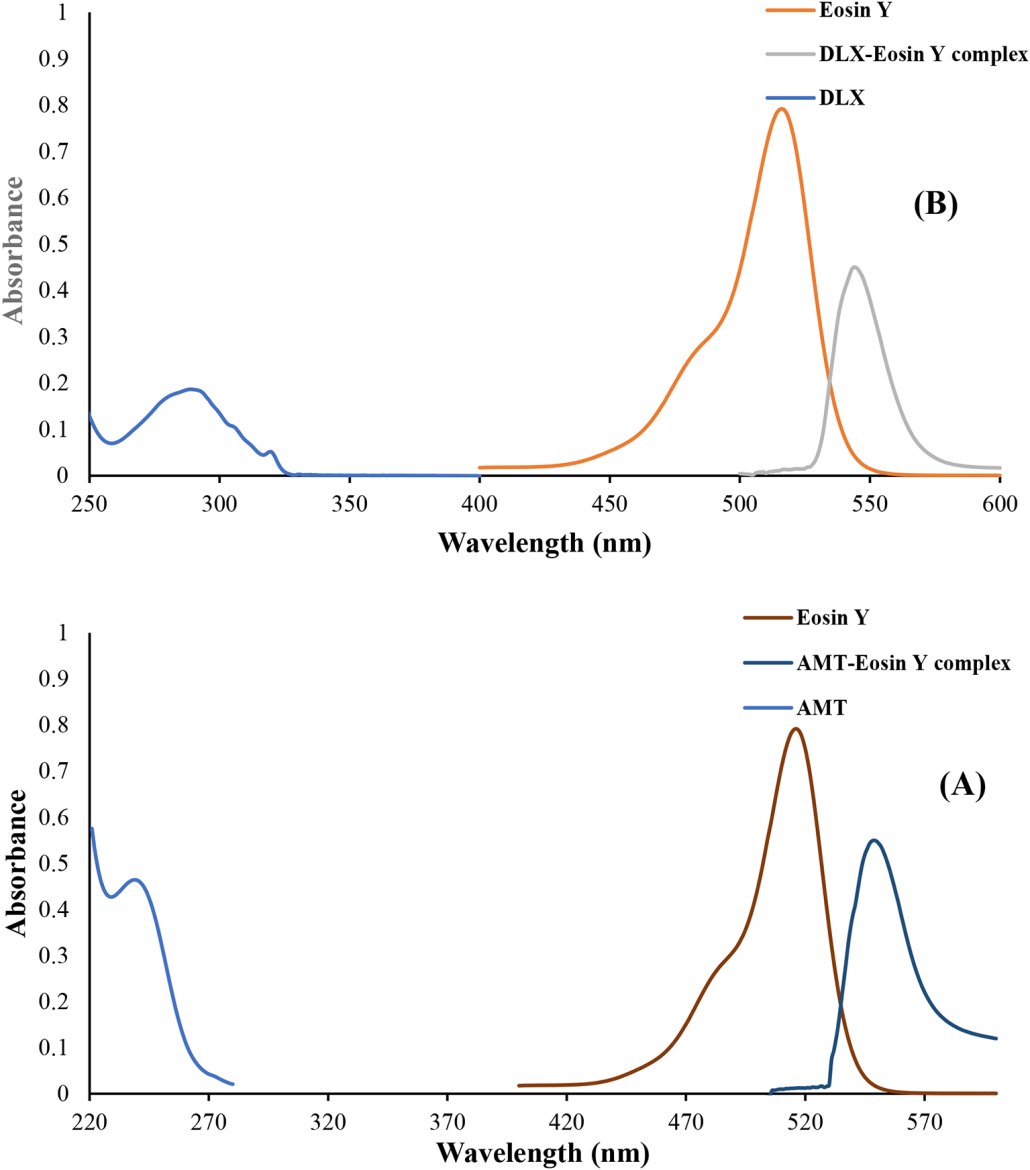
Absorption spectra of (4 µg mL^-1^) of DLX, (1 × 10^− 3^ M) eosin Y and its association complex with DLX (**A**), and (4 µg mL^-1^) of AMT, (1 × 10^− 3^ M) eosin Y and its association complex with AMT (**B**)

In order to produce stable, water-soluble ion pairs whose absorption could be precisely estimated, eosin Y was selected as the ion-pairing substance. The method outlined here has the benefit of being easy to use, quick, accurate, and precise in identifying DLX and AMT in dosage forms without any impact of common excipients. Additionally, it takes less time and doesn’t need laborious extraction processes or a variety of complex treatments. The method is also suited for routine analysis in quality control laboratories due to its satisfactory sensitivity and simplicity. The suggested approach is based on the formation of an ion association complex between eosin Y and the medications under study. These complexes most likely resulted from an electrostatic contact between the carboxylate anion of the dye and the amino group, which is the most basic center in the drug molecule. This mostly happens in an acidic solution, which causes eosin’s electron delocalization to increase and the dye to undergo a bathochromic shift of roughly 30 nm resulting in the formation of a new peak at 546 nm (Fig. 2). The proposed method is based on the formation of a water-soluble ion association complex between eosin Y and DLX and AMT.

### Experimental parameters optimization

The complex formation and stability influencing variables have been studied and optimized specifically.

#### Effect of pH

**Fig. 3:**
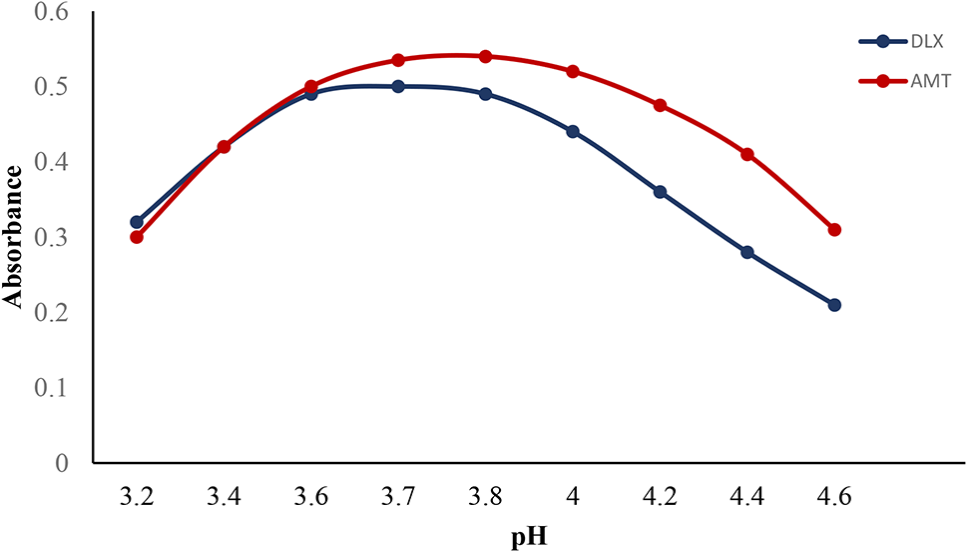
Effect of pH on the absorbance of the association complex formed between AMT (4 µg mL^-1^) and (4 µg mL^-1^) DLX with eosin Y (1 × 10^− 3^ M)

Since the investigated media needs to be sufficiently acidic to produce the ideal environment for the medication and eosin Y to combine and develop an ion pair interaction, the pH of the medium had been looked at as a crucial parameter. An acetate buffer with a pH range of 3.2–4.6 was used to investigate the impact of pH for AMT. It was detected that pH 3.8 produced the greatest absorbance. For DLX, Teorell-Stenhagen buffer was evaluated with pH that ranged from 3.2 to 4.6, with pH 3.7 being the proper value since it produced the maximum absorbance value. The outcomes were declined by increasing or reducing pH values, as indicated in Fig. 3. At pH 3.8 for AMT and pH 3.7 for DLX, the medication undergoes complete protonation, yielding the corresponding cations, (HDLX^+^) and (HAMT^+^). The medication undergoes complete protonation, yielding the corresponding cations, HDLX^+^ and HAMT^+^. The dye will also exist in some kind of monovalent anionic state. As a result, the medication cation and dye anion combine through the processes of electrostatic binding, generating ion pair complexes. The optimum pH value was 3.8 for AMT and 3.7 for DLX.

#### Buffer volume effect

**Fig. 4:**
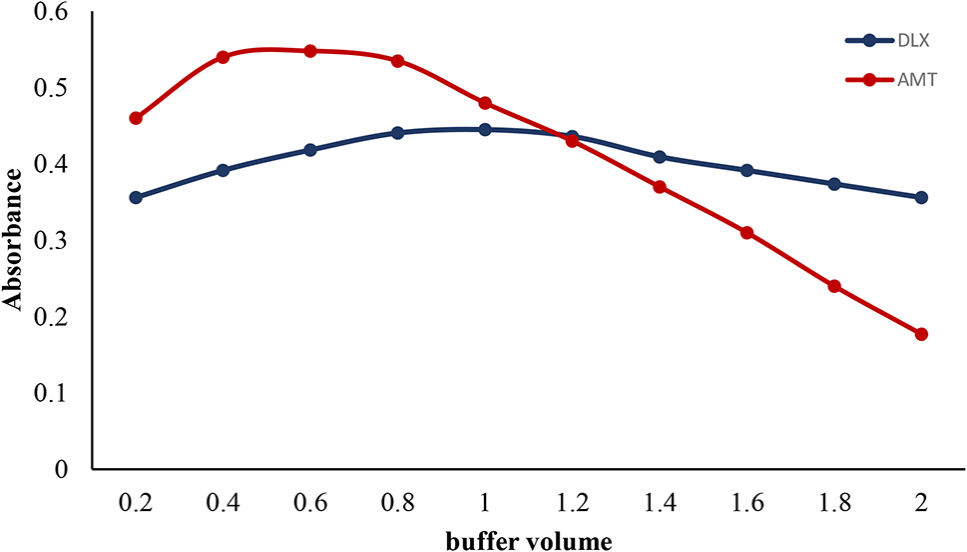
Effect of buffer volume on the absorbance of the association complex formed between AMT (4 µg mL^-1^) and (4 µg mL^-1^) DLX with eosin Y (1 × 10^− 3^ M)

The impact of buffer solution volume on the absorbance intensity was studied through the range of 0.2-2 mL of either Teorell-Stenhagen buffer for DLX or acetate buffer for AMT. It was noticed that 1 mL of Teorell-Stenhagen buffer was adequate to obtain the highest absorbance for DLX, whereas 0.6 mL of acetate buffer solution was recommended in the case of AMT, as indicated in Fig. 4. Lower volumes have produced lower results as the medium’s pH hadn’t been sufficiently adjusted (Fig. 4). The optimum volume was found to be 1 mL of Teorell-Stenhagen buffer for DLX and 0.6 mL of acetate buffer for AMT.

#### Volume of Eosin Y effect

**Fig. 5:**
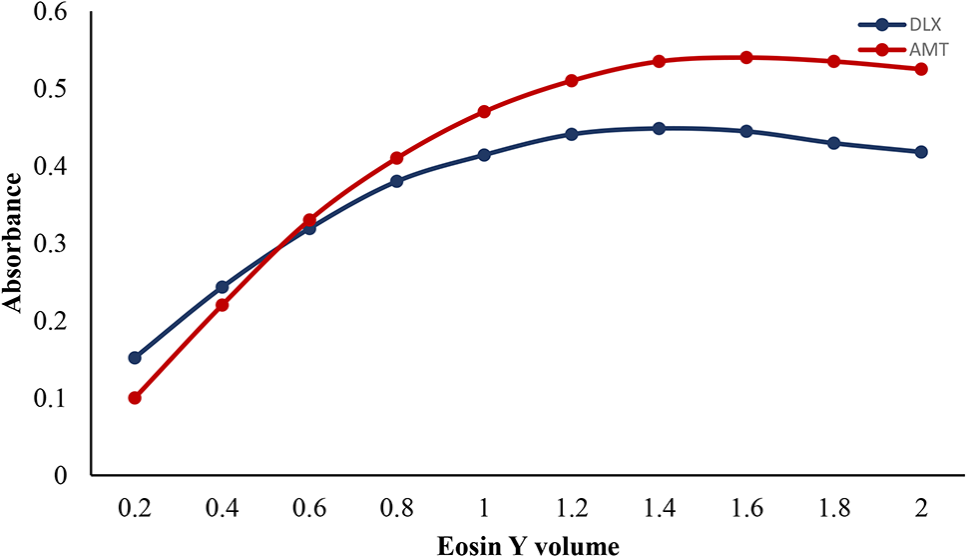
Effect of volume of eosin Y (1 × 10^− 3^ M) on the absorbance of the association complex formed with AMT (4 µg mL^-1^) and (4 µg mL^-1^) DLX

Several volumes of eosin Y (1 × 10^− 3^ M) were studied to determine the best volume of the dye. When eosin Y volume has been raised up to 1.2 mL for DLX and 1.4 mL for AMT, the absorption value obtained improved practically in a linear manner. There is no discernible variation was found until 1.6 mL for DLX and 2.0 mL for AMT. The highest reading for DLX was attained when 1.4 mL of eosin Y reagent was used while a volume of 1.6 mL of eosin Y was found to be the optimum for AMT, as indicated in Fig. 5. The reason for poor outcomes when utilizing smaller dye quantities is that it does not appear to be enough dye for the reaction to occur. The optimum volume of the dye was found to be 1.2 mL for DLX and 1.4 mL for AMT.

#### Effect of diluting solvents

**Fig. 6:**
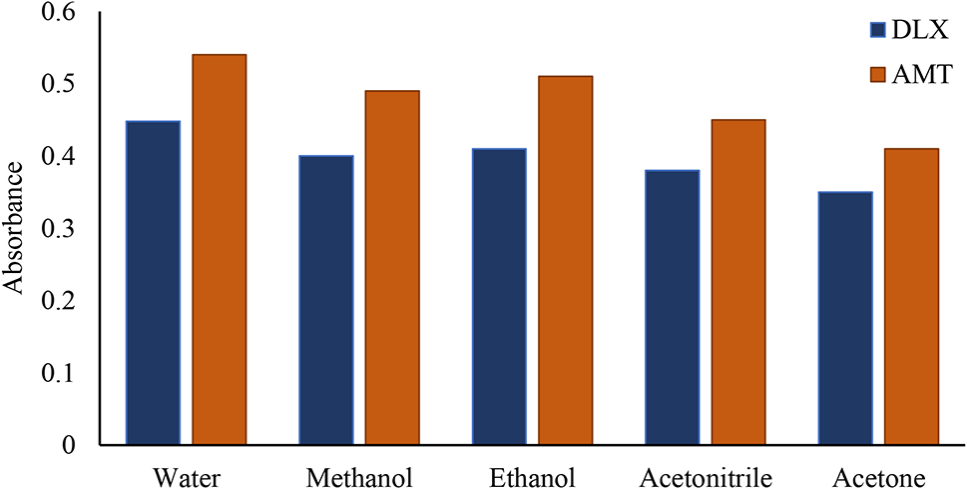
Effect of diluting solvent on the absorbance of the association complex of 4 µg mL^-1^ DLX or AMT with 1.0 × 10^− 3^ M eosin Y

A variety of solvents including acetonitrile, ethanol, methanol, water, and acetone were used to dilute the reaction solution. Water has been determined to be the most effective diluting solvent. As seen in Fig. 6, the remaining solvents yielded less favorable outcomes. Since water is inexpensive, readily available, and environmentally friendly, it was selected as the most suitable solvent.

#### Effect of addition order of reagents

The effect of addition order of the reagents on absorbance value of the system was also investigated. For this, the reagents were added in different sequences and absorbance value was measured. It was observed that there is negligible effect of the addition order of reagents on the absorbance intensity. The chosen sequence was: drug, buffer, eosin Y.

### Validation of the methods

#### Linearity and range

The calibration curve for the reaction of the medications under investigation with eosin Y was created under the indicated optimal reaction conditions. Eight concentrations of the standard solution of the investigated drugs were examined, and all measurements were carried out five times. A strong linear correlation was detected in the range of 0.5-8 and 1–7 µg mL^− 1^ for DLX and AMT, respectively. Table 1 summarizes the regression equation (Y = aX + b), where Y is the absorbance, X is the conc. (µg mL^− 1^), b is the intercept, a is the slope, and r is the correlation coefficient. The method’s good linearity was validated by the attained correlation coefficient’s closeness to unity (0.9996 for DLX and 0.9997 for AMT).

**Table 1:** Regression equation and validation parameters for the proposed spectrophotometric methods. LOD is limit of detection and LOQ is limit of quantitation

Parameters	Duloxetine	Amitriptyline
Linear range (µg mL^− 1^)	0.5-8	1–7
Linear regression equation	Y = 0.12X-0.05	Y = 0.14X-0.02
Slope (b)	0.12	0.14
Standard deviation of slope (S_b_)	0.001	0.0015
Intercept (a)	-0.05	-0.02
Standard deviation of intercept (S_a_)	0.006	0.0067
Correlation coefficient (r)	0.9996	0.9997
Determination coefficient (r^2^)	0.9992	0.9994
Number of determinations	5	5
LOD (µg mL^− 1^)	0.16	0.16
LOQ (µg mL^− 1^)	0.48	0.49

LOD = Limit of detection, LOQ = Limit of quantitation

#### Quantitation (LOQ) and detection (LOD) limits

The LOD and LOQ values which were determined based on the slope and the standard deviation of the intercept of calibration curve, were calculated in agreement with the ICH regulations [56]. The assay’s outstanding sensitivity is demonstrated by the following formulas: LOD = 3.3σ/slope and LOQ = 10σ/slope, with estimated LOD values were 0.16 µg mL^− 1^ for both drugs, while LOQ values were 0.48 and 0.49 µg mL^− 1^ for DLX and AMT, respectively.

#### Accuracy and precision

Four concentrations of the standard solution of the investigated drugs within the linear range were examined, and measurements were done in triplicate, in order to assess the approaches’ accuracy. The calculations showed respectable matches between measured and real outcomes, and the outcomes are revealed in Table 2 as % recovery ± SD. Three concentrations were used to examine the approach’s precision at low, middle, and high levels. The evaluation of intra-day precision involved analysis on the same day, whereas the evaluation of inter-day precision involved conducting assays on three successive days. The excellent precision is demonstrated by the low relative standard deviation values (Table 3). The % recovery values are near to 100% and SD and %RSD values are less than 2.

**Table 2:** Evaluation of accuracy of the analytical procedures by standard addition method

Drug	Amount taken of tablet extract (µg mL^− 1^)	Amount added of pure drug (µg mL^− 1^)	Amount found (µg mL^− 1^)	% Recovery͙͙* ± SD
DLX	2	0	1.99	99.33 ± 1.24
	2	1	3.01	100.19 ± 0.62
	2	2	4.04	101.12 ± 1.18
	2	3	4.99	99.85 ± 1.34
AMT	2	0	1.99	99.86 ± 0.74
	2	1	3.04	101.36 ± 1.09
	2	2	4.01	100.36 ± 1.14
	2	3	4.94	98.75 ± 0.97

*Mean of three determinations, SD, Standard deviation

**Table 3:** Evaluation of intra-day and inter-day precisions of the proposed methods

Drug	Conc. (µg mL^− 1^)	% recovery ± SD*			RSD*	
		Intra-day precision	Inter-day precision		Intra-day precision	Inter-day precision
**DLX**	2	98.67 ± 0.92		99.38 ± 1.33	0.93	1.34
	4	99.70 ± 0.70		99.86 ± 0.92	0.70	0.92
	8	101.92 ± 0.93		101.25 ± 1.05	0.91	1.04
**AMT**	2	101.14 ± 1.16		101.56 ± 1.35	1.15	1.33
	5	100.29 ± 0.96		100.32 ± 1.12	0.96	1.12
	7	100.65 ± 1.31		99.59 ± 1.55	1.30	1.56

*Mean of three determination, SD, Standard deviation

#### Ruggedness

To examine the ruggedness of the procedures, the intra-day precision and inter-day precision were evaluated. the precision of the proposed method is reasonably high, as indicated by the low values of the percentage relative standard deviation (%RSD).

#### Robustness

The impact of small changes in the variables being studied (pH, dye volume, and buffer volume) on the analytical efficiency of the approach was used to evaluate the robustness of the techniques. The attained results explained that minor differences did not meaningfully influence the procedure’s results in any of the studied parameters as the resulted %recoveries were close to 100% and SD did not exceed 2%. This explains the robustness of the recommended approach (Table 4).

**Table 4:** Robustness study of the proposed methods for determination of DLX (4 µg mL^-1^) and AMT (4 µg mL^-1^)

Parameter	Value		% recovery ± SD*	
	DLX	AMT	DLX	AMT
pH	3.4	3.7	101.91 ± 0.72	99.56 ± 1.20
	3.6	3.9	100.47 ± 0.64	98.46 ± 1.35
Volume of eosin (mL)	1.3	1.5	100.20 ± 1.43	99.25 ± 0.71
	1.5	1.7	101.26 ± 1.30	99.78 ± 0.61
Volume of buffer (mL)	0.9	0.5	101.85 ± 0.32	100.72 ± 0.49
	1.1	0.7	99.94 ± 1.21	99.28 ± 1.27

*Mean of three determination, SD, Standard deviation

### Stoichiometry of the reaction between Eosin Y and the cited drugs

**Fig. 7:**
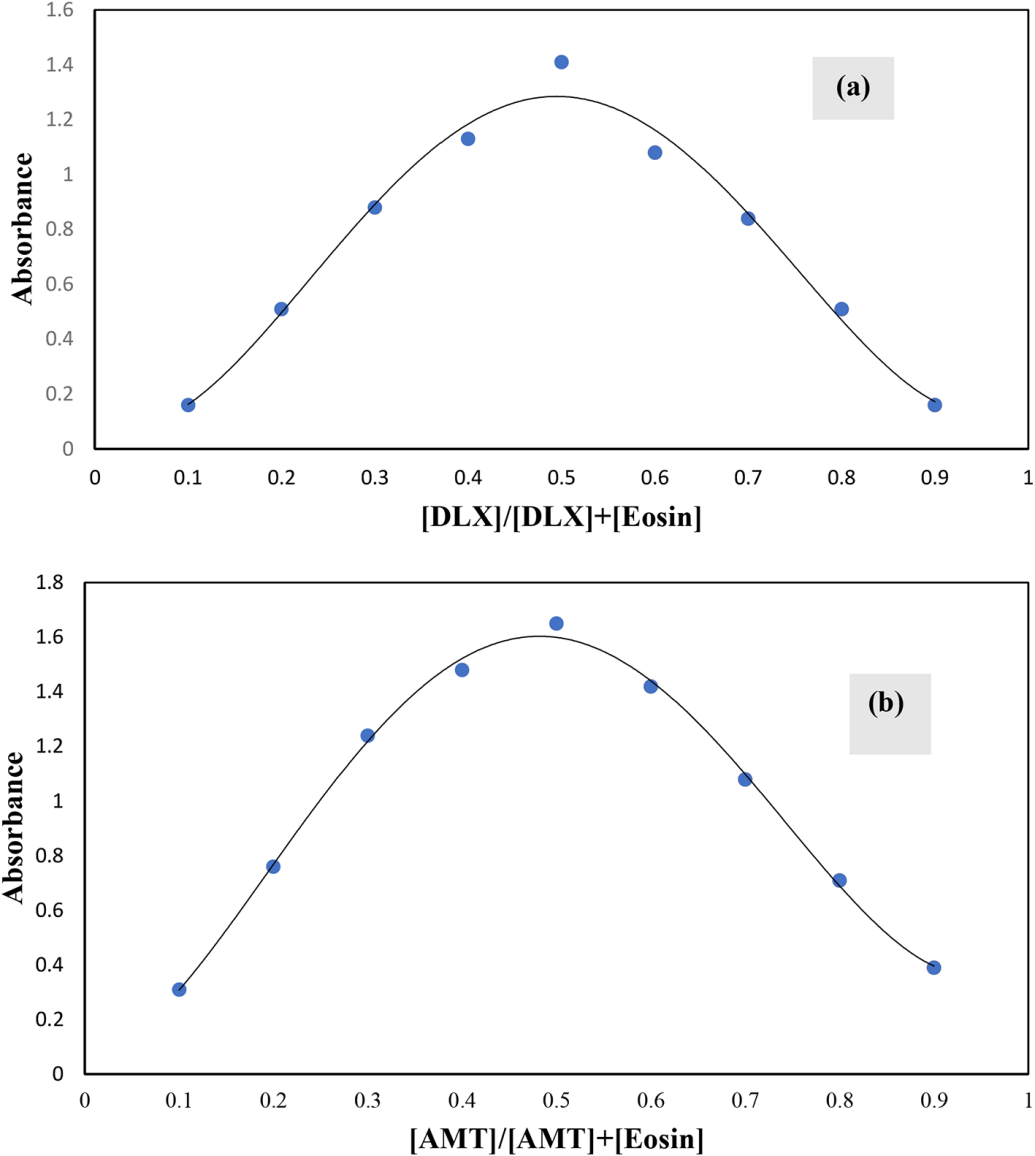
Job’s plots obtained for the reaction of eosin Y with DLX (**a**) and AMT (**b**) using equimolar concentration solutions (1 × 10^-4^ M)

The composition of the complex between eosin Y and cited drugs has been determined by applying Job’s method of continuous variation. Drug and dye solutions were made at an equimolar concentration of (1 × 10 − 4). Different mole fractions (0.1–0.9) of either drug and dye were mixed in complementary volumes totalling 1 ml into a 10 ml flask. Following the general methodology, the whole procedure had been completed. The measured value for each solution was adjusted regarding its blank reading. As shown in Fig. 7, the plots reached a maximum value at a mole fraction of about 0.5 for either of the studied drugs. This indicated the formation of 1:1 drug: dye complexes. In weakly acidic medium, eosin Y exists mainly in the monovalent anionic form (HR–). There are two possibilities for the ionization of eosin Y, by dissociation of the hydroxyl or carboxylic groups. It was suggested previously that the hydroxyl group tends to dissociate more easily than the carboxylic group. Therefore, the eosin Y monovalent anion is formed by the ionization of the hydroxyl group. The cited drugs have tertiary or secondary amino groups that are easily protonated in an acidic medium to form positively charged cations. The ion pair associate complex is formed by the interaction of the protonated amino group of studied drugs with the ionized hydroxyl group of the eosin Y mono anion through electrostatic attraction.

### Application

#### Application of pharmaceutical formulations

The present spectrophotometric techniques were used to analyze DLX in Cymbatex capsules and AMT in tryptizol tablets. Furthermore, previously reported approaches were used to analyze the dosage forms [7] for DLX and [26] for AMT, and the F-test and Student’s t-test were used for comparing the results of the suggested and reported methods. Table 5 displays no statistically significant differences between the suggested and published approaches, as the F-test and Student’s t-test values were not larger than those calculated at a 95% confidence level.

**Table 5:** Analysis of DLX and AMT in dosage forms by the reported and proposed methods

Dosage form	Proposed method	Reported method [7, 26]	t-test ^b^ value	F-test ^b^ value
	% recovery ± SD ^a^	% recovery ± SD ^a^		
Cymbatex 20 mg capsule	100.11 ± 0.44	99.48 ± 0.88	1.19	2.18
Tryptizol 10 mg tablet	98.92 ± 0.67	98.69 ± 1.33	0.18	1.88

^a^ Mean of five measurements

^b^ Tabulated value at 95% confidence limit, F = 6.338 and t = 2.306

#### Content uniformity testing

When the medication content in tablets or capsules is not more than 25 mg or its proportion is less than 25% of the contents of capsules or tablets, testing the drug homogeneity within the capsules is advised [57]. It may be hard and time-consuming to test for content homogeneity in each capsule or tablet. Consequently, the present work has the advantage of being a quick and easy assay because there is no need for extraction or heating, which takes time, because the complex forms instantly. These advantages make it possible to directly examine cymbatex capsules and tryptizol tablets. The acceptance value can be calculated using the following formula, as stated in US pharmacopoeia directives: AV=| M-$\:\stackrel{-}{X}$| + KS, where M is a reference value, K is the acceptability constant (which, in the instance of 10 capsules or tablets, is equal to 2.4), S represents the standard deviation, and $\:\stackrel{-}{X}$ is the average percentage of each material recovered. The AV need to be less than the highest AV that is permitted (L1 = 15). The value of will determine how the preceding equation is changed.

- M = $\:\stackrel{-}{X}$ (AV = KS) if 98.5% ≤ $\:\stackrel{-}{X}$≤101.5%.
- M = 98.5% (AV = 98.5–$\:\stackrel{-}{X}$ + KS) if $\:\stackrel{-}{X}\:$<98.5%.
- M = 101.5% (AV = $\:\stackrel{-}{X}$– 101.5 + KS) if $\:\stackrel{-}{X}$ >101.5%.

The homogeneity of cymbatex capsules and tryptizol tablets was demonstrated by the data in Table 6, since the acceptance value was below the upper limit permitted.

**Table 6:** Application of the proposed methods for the content uniformity test of cymbatex capsules and Tryptizol tablets

Tablet number	% Recovery	
	DLX	AMT
1	101.44	101.30
2	102.47	103.06
3	101.22	102.77
4	98.41	101.22
5	100.93	98.65
6	101.55	99.385
7	97.12	100.86
8	102.31	97.91
9	101.88	100.12
10	98.25	101.59
**Mean**	100.46	100.69
S*`	2.05	1.68
AV*	5.41	4.38
L1*	15	15

*S: Standard deviation, AV: Acceptance value, L1: Maximum allowed acceptance value

### Evaluation of the greenness of the methods

**Fig. 8:**
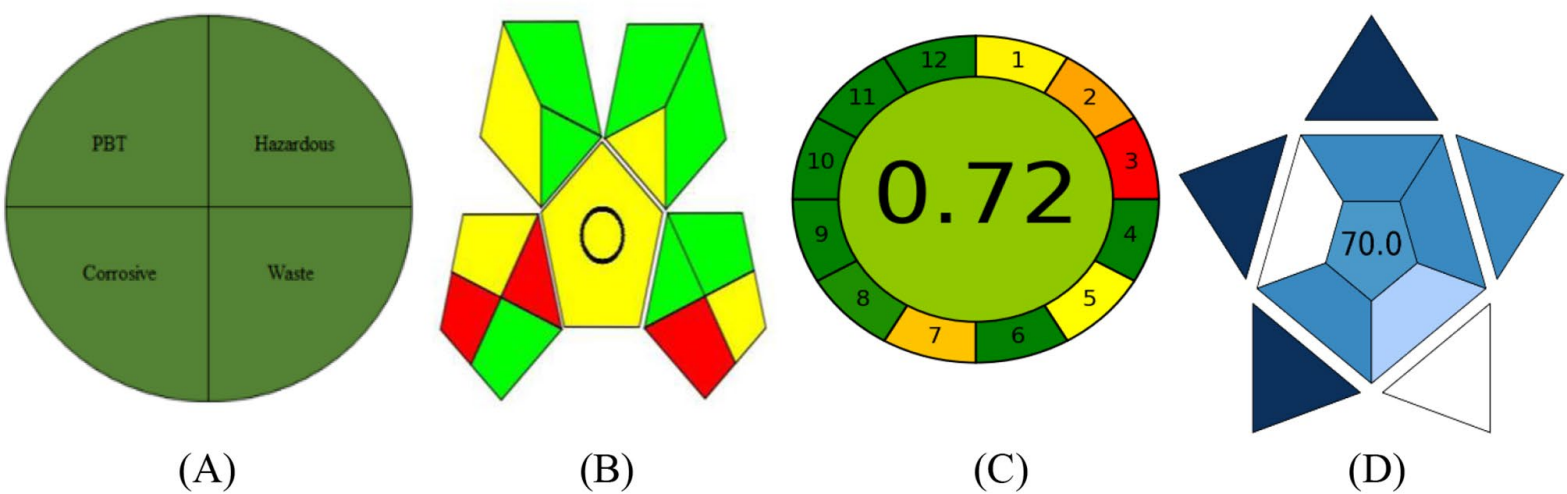
Evaluation of the greenness of the proposed spectrophotometric methods using NEMI (**A**), GAPI (**B**) and AGREE (**C**), and the blueness using BAGI (**D**) tools

Metrics related to green chemistry must be continuously developed and enhanced. An entirely green analytical technique will avoid the usage of hazardous solvents, high consumption of energy, chemical derivatization, and large waste output. The technique related to the NEMI was utilizes to evaluate the greenness of the proposed approaches [58, 59]. The greenness profile of the established technique was evaluated by the National Environmental Method Index (NEMI), and the method met al.l the criteria for being considered a green approach (Fig. 8). This was achieved by utilizing solvents that are non-persistent, non-bioaccumulative, and non-toxic (PBT). The recently proposed spectrophotometric approaches are dependent on methanol, which is not a PBT solvent. Additionally, the pH of the reaction was 3.8 for AMT and 3.7 for DLX, therefore not corrosive. The chemicals used in the method were non-hazardous, and the waste generated was minimal and did not exceed 50 mL. Based on these findings, the suggested method was deemed environmentally friendly, meeting all four quadrants of the greenness profile. As Fig. 8 illustrates, they can be considered as green approaches. More recently, the greenness of an analytical approach has been estimated using metrics such as the analytical Eco scale score [60]. The Eco-scale is a straightforward method used for the assessment of the analytical method greenness. The subsequent equation (analytical Eco-Scale score = 100 - total penalty) is used to calculate the total score. A penalty point was assigned for each procedure’s defined parameters, such as the number of chemicals used, dangers to employees, waste products, and consumption. After that, the total penalty was calculated by summation of these penalty points. If the score exceeds 75, the analytical method is considered green. Water is a non-toxic solvent that produced very little waste and required very little energy during the analysis. As a result, as indicated in Table 7, the evaluated technique obtained a high eco-scale score (95). This indicates that the technique has a high level of environmental friendliness.

**Table 7:** Evaluation of the greenness of the proposed methods using eco scale method

Item	Parameter		PPT score
Technique	Spectrophotometry		0
Reagent	Eosin Y		1
Amount of reagent	< 10 mL		1
Solvent(s)	Water	LSH	0
Heating	—		0
Temperature	25 °C		0
Cooling	—		0
pH	3.7		0
Energy (kWh per sample)	< 1.0		0
Occupational hazards	(Analytical process hermitization)		0
Waste	(1–10 mL)		3
Total penalty points			5
analytical eco-scale total score^a^			95

^a^ If the score is greater than 75, it represents excellent green analysis. If the score is greater than 50, it represents acceptable green analysis. If the score is less than 50, it represents inadequate green analysis. LSH: less severe hazard, PPT: penalty point

Green Analytical Procedure Index (GAPI) can be used to assess how environmentally friendly an analytical process is, from collecting samples to final analysis. GAPI evaluates various aspects of the analytical process, the greenness of each stage in the analysis process is evaluated, and the result is expressed as a pictogram. The five major pentagrams that make up the GAPI pictogram are separated into fifteen sections, each of which focuses on an analytical stage. In order to evaluate the ecological implications, GAPI uses green, yellow, and red colors. Red indicates negative impact, whereas yellow indicates medium, and green indicates low impacts on the environment [57, 61]. The suggested technique revealed 5 yellow, 7 green, and 3 red shaded fields when evaluated using the GAPI metric. These results indicate the high greenness of the proposed method.

The most recent metric is the Analytical Greenness Metric Approach and Software (AGREE). The 12 principles of greenness are referred to in the input parameters, the AGREE diagram is made up of an outer boundary of 12 sections, each of which indicates one of the principles of green analytical chemistry. The AGREE uses a color scale (green, yellow, and red) and a score (0–1) to indicate the efficiency with which each analytical step is performed; every one of the twelve parameters is converted into the typical 0–1 range scale [52, 62]. The sum of the evaluation results for every principle determines the overall evaluation value. The result is a clock-like diagram with the total rating and color representation in the center of the figure. The evaluation can be done with free software, which generates a report and an auto-generated graph. Free software that creates an auto-generated graph and a report is available for assessment. The AGREE assessment indicates that the proposed method’s 0.75 score is dependent on various factors, including the type of solvent and the amount employed, as well as the potential harm of the solvent to humans and the surroundings. Thus, the recommended method has little effect on the environment, as shown in Fig. 8.

### Blueness evaluation

To evaluate the analytical procedure’s practical aspects, an innovative metric called the BAGI has been developed [63, 64]. Two groups of findings are obtained by the BAGI metric tool: a numerical score located in the center of the pictogram and a graphical depiction shaped like an asteroid. The evaluation result is visually represented by the asteroid-shaped pictogram, which is composed of many blue color tones that indicate different levels of compliance (dark blue for high, blue for moderate, light blue for low, and white for non-compliance). Ten factors are taken into account by BAGI in order to produce a pictogram and a score that demonstrate the utility and efficacy of an analytical procedure (Table 8). In order for the analytical process to be considered practical, it is recommended that the final score be higher than 60. The ultimate rating, which is displayed in the center of the pictogram (Fig. 8), gives the recommended strategy an overall score of 80.

**Table 8:** The 10 factors utilized in the evaluation of the proposed methods using the BAGI

Parameter	Rating	Remarks
1. Type of Analysis	moderate blue	The method is categorized as quantitative.
2. Multi-Analyte Procedure	white	One component is determined by the approach
3. Analytical Technique Used	moderate blue	A spectrophotometer device was used, which is easily accessible in most labs.
4. Simultaneous Sample Preparation	light blue	The suggested method’s simultaneous preparation’s ease of use and time-saving nature.
5. Sample Preparation	moderate blue	It involves little and no payment for sample preparation.
6. Samples Per Hour	dark blue	
7. Availability of Reagents	dark blue	there are no derivative reagents—common reagents that are sold commercially
8. Preconcentration	dark blue	it doesn’t require preconcentration.
9. Automation of Device	white	Procedures with manual devices
10. Amount of Samples	moderate blue	The sample volume is small and the direct fluorometric technique

## Conclusion

A spectrophotometric approach that is simple, green, sensitive, accurate, and precise was created to determine the amount of DLX and AMT in pharmaceutical formulations, with a detection limit of 0.16 µg mL^− 1^. In order to produce stable and water-soluble ion pairs whose absorbance could be precisely estimated, eosin Y was selected as the ion-pairing substance. The suggested approach has numerous benefits, including being suitable, easy to use, low in time, and not requiring a lot of complex treatments or laborious extraction processes. It also has acceptable sensitivity and repeatability. The technique was effective in detecting the medications under investigation in various dosage forms and assessing the consistency of content. The method’s greenness was evaluated using a variety of cutting-edge technologies, and the findings showed a high greenness rating. As a result, drug companies as well as quality control institutions can assess medications using this technique. In the future, the suggested procedure may be applied for the determination of other drugs using the same procedure and reagent as the procedure is simple and the dye is affordable and water-soluble. Also, the environmental impact may be evaluated using more advanced and new tools.

## Data Availability

The datasets used and/or analyzed during the current study are available from the corresponding author on reasonable request.
